# Autophagy as a therapeutic target in pancreatic cancer

**DOI:** 10.1038/s41416-020-01039-5

**Published:** 2020-09-15

**Authors:** Max Piffoux, Erwan Eriau, Philippe A. Cassier

**Affiliations:** 1 Department of Medical Oncology, Centre Léon Bérard, Lyon, France; 2 INSERM UMR 1197—Interaction cellules souches-niches: physiologie, tumeurs et réparation tissulaire, Villejuif, France; 3 Laboratoire matière et systèmes complexes, Université de Paris, Paris, France; 4 Team 11 « Metabolism, Cancer, Immunity », UMR S1138, Centre de Recherche des Cordeliers, Paris, France; 5 TGFβ and Pancreatic Cancer Lab, UMR INSERM 1052 - CNRS 5286, Centre de Recherche en Cancérologie de LYON (CRCL), Centre Léon Bérard, Lyon, France

**Keywords:** Pancreatic cancer, Pancreatic cancer

## Abstract

Pancreatic ductal adenocarcinoma (PDAC) is characterised by early metastasis and resistance to anti-cancer therapy, leading to an overall poor prognosis. Despite continued research efforts, no targeted therapy has yet shown meaningful efficacy in PDAC; mutations in the oncogene KRAS and the tumour suppressor TP53, which are the most common genomic alterations in PDAC, have so far shown poor clinical actionability. Autophagy, a conserved process allowing cells to recycle altered or unused organelles and cellular components, has been shown to be upregulated in PDAC and is implicated in resistance to both cytotoxic chemotherapy and targeted therapy. Autophagy is thus regarded as a potential therapeutic target in PDAC and other cancers. Although the molecular mechanisms of autophagy activation in PDAC are only beginning to emerge, several groups have reported interesting results when combining inhibitors of the extracellular-signal-regulated kinase/mitogen-activated protein kinase pathway and inhibitors of autophagy in models of PDAC and other KRAS-driven cancers. In this article, we review the existing preclinical data regarding the role of autophagy in PDAC, as well as results of relevant clinical trials with agents that modulate autophagy in this cancer.

## Background

Pancreatic ductal adenocarcinoma (PDAC), characterised by early metastasis and poor prognosis (the mortality to incidence ratio is 94%),^1^ is projected to become the primary cause of cancer-related deaths in the USA by 2030.^2^ Most patients present with advanced disease and the majority of the 20–25% of patients with operable disease will relapse. In the metastatic setting, the outcome remains poor despite improvements in chemotherapy regimens (FOLFIRINOX/gemcitabine-nab paclitaxel), with a 5-year survival rate of just 3%.^3^ KRAS, TP53, CDKN2A and SMAD4 are the most commonly altered genes in human PDAC, but are considered poorly therapeutically actionable in most cases; indeed, no targeted therapy is yet approved for PDAC and trials have so far been underwhelming despite strong investments in the development of KRAS pathway inhibitors.^4^ Immune checkpoint inhibitors are also considered ineffective^5^ for PDAC, apart from some rare cases of the disease that harbour microsatellite instability (MSI-H PDAC).^6,7^ Thus, there is an urgent need to investigate further means by which PDAC can be therapeutically targeted.

Autophagy is an evolutionarily conserved physiological process induced by nutrient depletion^8^ or cell stress, which leads to the recycling of intracellular compounds, mostly through the engulfment of a portion of the cell cytosol (including organelles), in vesicles that fuse with lysosomes for subsequent degradation, yielding new metabolites— building blocks—to fuel cellular metabolic and energy pathways.^9^ Additional details on the general mechanisms of autophagy are depicted in Box 1 and have been reviewed elsewhere.^10–12^ In this review, we will use the term autophagy as a proxy for macroautophagy—that is, the engulfment of cell cytoplasm in autophagosomes, which is the best-known autophagy mechanism. A large variety of organelle- or compound-selective mechanisms have been described, but will not be discussed in detail here.

In oncology, autophagy has mainly been described as a mechanism of resistance to various cancer treatments such as chemotherapy,^13^ targeted therapy^14^ or immunotherapy.^15^ The role of autophagy in cancer is complex and only partially understood, which has consequently slowed the development of agents targeting autophagy for the treatment of cancer. Interestingly, autophagy was first described as a tumour-suppressive mechanism, based on the fact that heterozygous deletion of mammalian Beclin1 (the orthologue of Atg6) led to the development of malignant neoplasms in various organs in mice.^16,17^ However, this partial autophagy deletion phenotype was not seen in mouse models that were totally autophagy deficient (in ATG5^−/−^ mice, for example):^18^ these mice spontaneously developed only benign liver tumours. One explanation for this unexpected observation could be that autophagy is a relatively weak tumour suppressor yet at the same time is required for the progression of benign tumours to malignancy.^18^ A dual role for autophagy in cancer initiation and progression might also be proposed based on its role in mitigating cell (including DNA) damage on the one hand, which might be important for suppressing tumour initiation, while on the other hand, its role in energy homoeostasis could help aggressive cancer cells to survive and grow in a stressed microenvironment. Alternatively, these different phenotypes might be explained by numerous autophagy-independent functions of the autophagy machinery: indeed, Beclin-1 has been shown to be involved in cell death, which could explain the tumour-promoting effect of the heterozygous loss of BCLN1 not observed with the deletion of other autophagy-related genes.^19^

In this article, we will review the roles of autophagy in tumour versus stroma, autophagy and metabolism, and autophagy and the immune response, as well as the available evidence to support the modulation of autophagy in PDAC, as well as clinical results obtained so far using inhibitors and activators of this cellular process.

Box 1: Overview of autophagy and its main effectors Autophagy can be induced to clear the cytoplasm of damaged components (such as protein aggregates or damaged mitochondria), but is mostly activated in order to maintain intracellular homoeostasis in the case of stress and damage, allowing cells to mobilise stock via the catabolism of intracytosolic components. UNC51-like kinase 1 (ULK1) and its initiation protein complex are regulated by its negative regulator mammalian target of rapamycin complex 1 (mTORC1) and its positive regulator AMP-activated kinase (AMPK), both of which are metabolic sensors. The phosphatidylinositol 3-kinase (PI3K) class III nucleation complex, whose best characterised protein is Beclin 1 (BECN1), is activated next, to induce, with the help of ATG9-bound vesicles, the nucleation of a pre-phagophore. LC3-II (ATG8) protein, a receptor that binds to LC3-interacting motifs (LIRs) on autophagosome cargos, is recruited onto the pre-phagosome through lipidation. This conjugation (with phosphatidylethanolamine) is performed by ATG4B, ATG3 and ATG7 with the help of the ATG12-conjugation complex and the phosphatidylinositol 3,4,5-triphosphate (PIP3) binding complex, which comprises WD repeat-domain phosphoinositide-interacting proteins (WIPIs) and zinc-finger FYVE domain-containing protein 1 (DFCP1). In a first step, LC3 is cleaved by ATG4B to reveal a glycine residue (creating LC3-I) which binds to ATG7 (E1-like enzyme shared with ATG12), and is transferred to its specific E2-like enzyme ATG3, and conjugated to the head group of phosphatidylethanolamine (PE) (creating LC3-II). ATG12 is activated by ATG7 and forms sequential intermediates with ATG7 and ATG10 (E2-like enzyme) before being conjugated with ATG5. ATG12-ATG5 conjugates act as E3-like enzymes to promote ATG8–PE conjugation. ATG16L is not required for the chemical reaction but ensure the lipidation occurs in the right membrane. These events lead to elongation and eventual closure of the phagophore to form the autophagosome. Once closed, the autophagosome’s double bilayer will undergo fusion with lysosomes to initiate degradation of the inner membrane and intra-autophagosome cargo. This step is mediated by SNARE-related proteins. Autophagy can be modulated by various drugs; the most frequently reported are depicted in green (activators) and red (inhibitors). Potential pharmaceutical targets are depicted in blue.

## Increased autophagy in PDAC

A high basal rate of autophagy has been described in several human PDAC cell lines, and autophagy was shown to be upregulated in the later stages of the progression of pancreatic intraductal neoplasia (premalignant lesions; PanIN) to PDAC, but not in normal pancreatic ducts.^20^ Inhibition of autophagy in these cases (using either pharmacological inhibition with chloroquine or siRNA-mediated knockdown of ATG5) reduced cell proliferation in vitro and tumour growth in vivo, in nude mice. The molecular mechanisms underlying these observations are still incompletely understood, and are somewhat specific to PDAC, as these effects were not observed in breast or lung cancer cell lines.^20^ Bardeesy and colleagues have proposed a model that might apply to PDAC: they suggest that a transcriptional programme leading to the increased expression of genes implicated in autophagy and lysosome function is driven by elevated expression of the microphtalmia transcription factor (MiTF) family members MiTF, TFE3 and TFEB.^21^ The increased expression and nuclear retention of MiT/TFE was shown to be a consequence of dysregulated nucleocytoplasmic transport by Importins, rather than a lack of cytoplasmic regulation by the nutrient sensor mammalian target of rapamycin complex 1 (mTORC1; phosphorylation by mTORC1 usually retains these proteins in the cytoplasm), and occurs in a nutrient-independent manner, indicating that the usual regulation of autophagy by nutrient sensing is bypassed in these PDAC cells. Settembre et al. showed in HeLa cells that the nuclear translocation of TFEB during starvation was inhibited by extracellular-signal-regulated kinase (ERK)/mitogen-activated protein kinase (MAPK) 2-mediated phosphorylation on serine-142.^22^ These findings were also observed in PDAC models, in which inhibition of the RAS–RAF–MEK–ERK/MAPK signalling cascade (where MEK is MAPK kinase) led to increased autophagic flux.^23,24^ Thus, the current picture suggests that increased autophagy in PDAC occurs despite the constitutive activation of ERK/MAPK signalling resulting from mutation of KRAS (see next paragraph): constitutive activation of ERK/MAPK signalling would be expected to lead to cytoplasmic retention of TFEB/TFE3 (due to phosphorylation by ERK/MAPK2) and decreased expression of autophagy/lysosome genes.^25^ Therefore, although ERK/MAPK signalling is clearly involved in autophagy regulation, it is unlikely that constitutive ERK/MAPK activation is the cause of increased baseline autophagy in PDAC. Other mechanisms in addition to increased nuclear MiT/TFE retention concur to increase autophagy in PDAC, as demonstrated by Wong et al., who showed that increased activity of the phosphatase PP2A towards UNC51-like kinase 1 (ULK1), an enzyme involved in initiating autophagy, was also responsible for increased autophagy in PDAC cells.^26^

## KRAS, TP53 and the role of autophagy in pancreatic carcinogenesis

Hotspot mutations in the KRAS oncogene are the most common genomic event in PDAC and are found in ~90% of human PDAC samples, while loss of the functional p53 tumour suppressor (in most cases by point mutations) occurs in ~75% of human PDAC samples.^27–29^ These genomic alterations are recapitulated in some of the most commonly used genetically engineered mice models of PDAC, which are based on the cre/lox-mediated expression in pancreatic tissue of mutant KRAS together with the loss of either TP53 (single allele mutation *TP53*^*R172H*^ or *TP53*^*+/*^) or the CDKN2A/INK4 locus.^30^ In these models, lesions usually appear when the remaining functional copy of TP53 or CDKN2A is lost (loss of heterozygosity), a phenomenon thought to be similar to the malignant transformation of PanIN lesions in human pancreas.^20^

Many studies have investigated the role of autophagy in KRAS and/or TP53-mediated carcinogenesis in different models, including pancreatic cancer, with somewhat conflicting results.

### A role for autophagy in PDAC progression

In a KRAS-driven lung cancer model, Guo et al. found that genetic ablation of Atg7 reduced tumour burden due to reduced proliferation of tumour cells; however, the *Atg7*^*−/−*^ mice died of lung inflammation.^31^ In a similar lung cancer model, genetic ablation of Atg5 in pneumocytes in an oncogenic Kras^G12D/+^ background led to an increase in tumour initiation (with higher numbers of benign hyperplastic lesions), but a decrease in the rate of progression to malignancy (decreased numbers of adenocarcinomas).^31,32^ Interestingly, Rao et al. showed that the initial increase in hyperplastic lesions was due to an increase in the number of regulatory T cells, which leads to a decrease in the immune-mediated clearance of abnormal hyperplastic cells. Rosenfeldt et al. reported similar findings in *Atg7*-deficient *Kras*^*G12D/+*^ and *Atg5*-deficient *Kras*^*G12D/+*^ mice (driven by the Pdx promoter).^33^ Furthermore, the homozygous deletion of *Trp53* in this model led to PDAC formation irrespective of autophagy, and shorter survival (due to tumour progression) in autophagy-deficient mice (both with *Atg5*^*−/−*^ and *Atg7*^*−/−*^ mice). In this study, pharmacological inhibition of autophagy with hydroxychloroquine also accelerated tumour progression in *Kras*^*G12D/+*^ *Trp53*^*−/−*^ mice.^34^ Thus, the authors concluded that p53 was a master regulator of the autophagy effect. This model has significant caveats, however: the pancreas develops in these mice without any functional p53, which probably affects their autophagy programme from the start and is different from the stepwise progression from PanIN to PDAC observed in other models and in humans.^12^ Also, other groups have not been able to replicate these results, and failed to show increased tumour formation in KRAS-mutant cell lines when autophagy was inhibited. For example, Eng et al.^33^ demonstrated using a large panel of cancer cell lines that autophagy was not protective against a wide variety of clinically active anti-cancer drugs and that the observed effect of chloroquine on tumour cells was not dependent on autophagy. It is important, however, to note that this study was carried out under very different conditions to the previously described ones. First, it used cell lines derived from fully transformed cancer tissue, which might have had additional genomic alterations reducing their dependence on autophagy, instead of GEMM. Secondly, the experiments were done in nutrient-replete conditions, which could significantly alter the requirement for autophagy, especially compared with the nutrient-poor conditions usually seen in pancreatic tumour. Finally, the in vivo experiments, which were done primarily in immunocompromised hosts, might not take into account the effects of autophagy-deficient tumour cells on innate and adaptive immune responses.^12,35^

Another study^36^ used a mouse model of pancreatic cancer comprising a *Kras* mutant with loss of a single *Trp53* allele as a more relevant model of human PDAC (as *Kras*^*mut*^*/Trp53*^*null*^ models lead to non-metastatic disease) to explore the role of autophagy in PDAC. This model showed that tumour-specific autophagy inhibition (by conditional *Atg5* knockdown) leads to an increase in the number of PanINs, but that these are unable to progress to invasive cancer. It also confirmed that treatment with chloroquine or hydroxychloroquine of murine cell lines (independent of their *Trp53* status) and various patient-derived xenograft models (in immunocompromised mice) led to decreased proliferation, increased DNA damage and apoptosis.

Consistent with these findings, White and colleagues^37^ showed, using an inducible *Atg7* deletion in a *Kras*^mut^/*Trp53*^null^ lung cancer model, that distinct phenotypes were observed depending on whether autophagy was inhibited during or after tumour initiation. Inhibition during tumour initiation did not alter the formation of tumours, but slowed tumour growth over time. By contrast, inhibiting autophagy after the initiation of tumours led to a block in tumour progression and the emergence of benign tumours (oncocytomas) rather than malignant tumours (carcinomas), leading to an overall reduced tumour burden.^37^ Overall, these studies, which support the role of autophagy in PDAC progression, prompted the launch of clinical trials testing the effect of chloroquine and hydroxychloroquine in patients with PDAC, the results of which will be discussed later in this review (see below).

### Can inhibiting autophagy promote carcinogenesis?

Interestingly, impaired autophagy was described as a cancer-promoting phenomenon by Todoric et al.,^38^ who showed that chronic stress, a known risk factor for PDAC in humans, impaired autophagy in the pancreas, leading to the subsequent induction of MDM2. MDM2 then mediates p53 degradation, leading to the transformation of benign lesions into PDAC. It should be noted, however, that this study does not provide definitive proof for the role of autophagy due to multiple alternative explanations to these observations.

A 2019 study by Görgülü et al. investigated the impact of *Atg5* gene dosage in a  *Kras*-mutant model of pancreatic cancer.^39^ Homozygous deletion of *Atg5* led to enhanced acinar-to-ductal metaplasia, but lesions failed to progress to high-grade PanIN lesions and PDAC. By contrast, single-allele knockout of *Atg5* led to increased malignant tumour formation and metastatic dissemination compared with mice with wild-type Atg5. Similar results were obtained using *Atg5* shRNA-mediated knockdown in cell lines. The authors found that *Atg5*^*+/−*^*/Kras*^*mut*^ cells were resistant to autophagy regulation (induction and inhibition) and displayed mitochondrial dysfunction and increased expression and secretion of protumorigenic cytokines, which led to increased tumour infiltration by M2 macrophages.^39^ Whether all these properties are due to constitutive activation of autophagy or whether this is due to an Atg5-related effect, however, remains unclear for two reasons: first, the effect on autophagy seems at best moderate in this model; and second, no other autophagy gene was tested. Notably, an increased number of M2 macrophages has also been reported by other groups studying autophagy in PDAC,^38,40^ and will be discussed later in this review.

## Autophagy in tumour cells versus stromal cells

Overall, although activation of autophagy is frequently observed in PDAC and other tumour types with oncogenic activation of the ERK/MAPK pathway,^41^ the dependency of KRAS-mutant tumours on autophagy seems model- and tumour-type dependent. In addition, one of the questions that has emerged from the modulation of autophagy in the different genetically engineered mouse models is the role played by autophagy in tumour cells versus the role in stromal/supporting cells. This issue is therapeutically relevant because, in most instances, anti-cancer drugs are given systemically (either orally or by intravenous or subcutaneous injection) and act systemically (i.e. on the whole organism) rather than targeting specific cell types, which might be desirable in the case of autophagy.

### A mouse model enabling the acute and reversible inhibition of autophagy

**Fig. 1:**
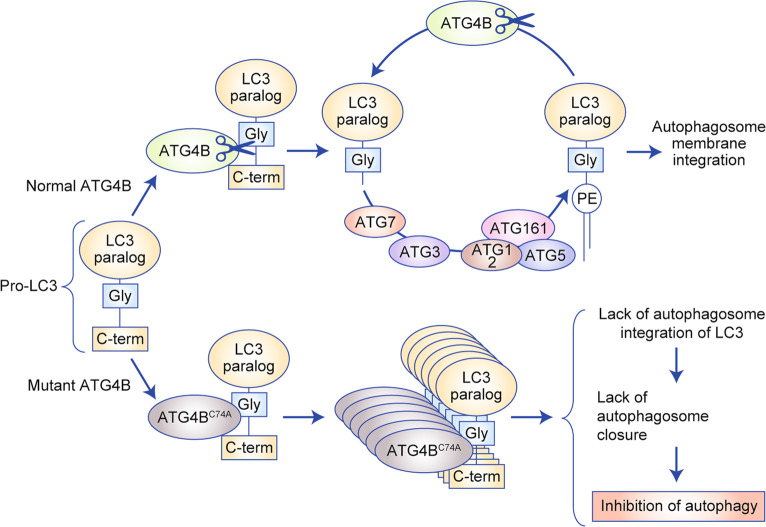
Mechanism of the dominant effect of mutant ATG4B (ATG4B^C74A^). Unlike normal ATG4B, ATG4B^C74A^ is devoid of catalytic activity and thus unable to process LC3 to LC3-I, but remains bound to unprocessed LC3, thus functionally sequesters it and prevents autophagosome closure and thus inhibits autophagy.

Yang et al. addressed this point by developing a mouse model expressing a CRE-inducible dominant-negative form of ATG4B (ATG4B^C74A^).^40^ This form of ATG4B interacts with the autophagy component LC3 but sequesters it, thus preventing its appropriate lipidation and incorporation into the autophagosome membrane (Fig. 1); the inhibition of autophagy can, therefore, be attained acutely and reversibly.^40,42^ Accordingly, crossing this strain with a mouse model of PDAC (*LSL-Kras*^*G12D*^, *Trp53*^*lox/+*^, *p48*^*Cre+*^) facilitates the inducible expression of the dominant-negative form in the pancreas: once the mice have developed established tumours, doxycycline can be used to induce expression of ATG4B^C74A^. Although mice that had tumours with a single ATG4B^C74A^ allele lost this mutant allele to regain normal autophagy, mice that had tumours with two ATG4B^C74A^ alleles showed complete tumour regression; as previously observed, however, extensive pancreatic metaplasia occurred. The authors demonstrated that metaplasia was not induced by the loss of ATG4B when mutant Kras was not expressed in pancreatic tissue, which is an important finding for the clinical translation of the autophagy inhibition strategy in PDAC. Interestingly, the authors found that intermittent autophagy inhibition (using intermittent doxycycline administration) maintained anti-tumour efficacy and improved the long-term survival of mice (no pancreatic metaplasia was observed).

### Autophagy maintains PDAC through tumour-cell-intrinsic and -extrinsic mechanisms

**Fig. 2:**
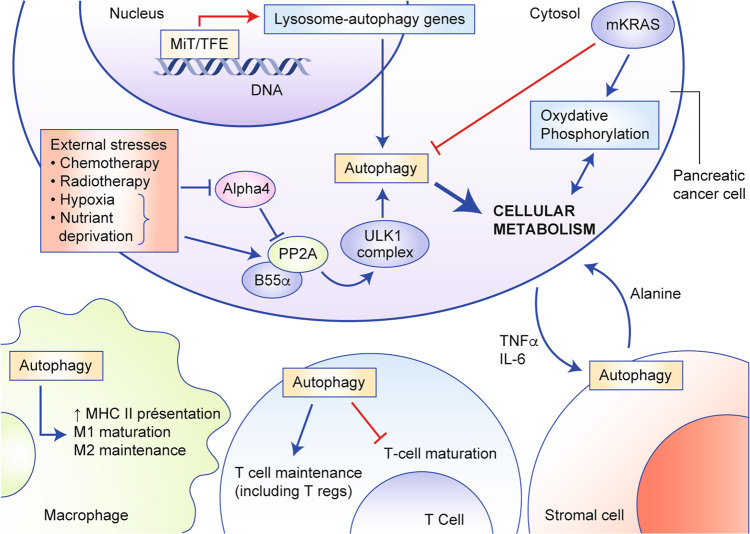
The various roles of autophagy in pancreatic cancer. The roles of autophagy in pancreatic tumour cells as well as in the components of the tumour microenvironment are depicted (red arrows indicate activation, blue bars indicate inhibition of cellular processes). Autophagy is activated by multiple cues in pancreatic cancer cells: increased autophagy gene expression upon transactivation by members of the MiTF/TFE3 transcription factor family, but also as a response to external stressors such as cytotoxic drugs, radiation, hypoxia, nutrient deprivation. Autophagy fuels the increased metabolism of tumour cells by providing energy and building blocks to help sustain proliferation. Cytokine secretion by cancer cells instruct normal stromal cells in the tumour microenvironment to release amino-acids such as alanine in the vicinity of tumour cells which provide additional nutritional support to proliferating cancer cells. Autophagy also plays a role in the maturation and maintenance of T cells as well as in antigen presentation by macrophages and other antigen-presenting cells.

Having demonstrated the efficacy of their model, the authors set out to assess the relative contribution of tumour–cell autophagy versus stromal autophagy (tumour–cell-autonomous and tumour–cell-nonautonomous mechanisms) in supporting tumour growth, as the same team had previously reported an important contribution of autophagy in non-cancerous pancreatic stellate (stromal) cells in supporting PDAC tumour cell growth.^43^ Their study used mouse cell lines grown in immunocompromised and immunocompetent mice to reveal two interesting findings: first, the effect of autophagy inhibition was more profound in immune-competent hosts, and relied on macrophage infiltration in this model; and, second, tumour–cell-autonomous and host-level autophagy inhibition both influenced tumour growth. The growth-supporting effect of host autophagy has also been described in other models and seems to rely on the secretion of cytokines such as tumour necrosis factor α (TNFα) and interleukin (IL)-6 by nutrient-stressed tumour cells^44^ (Fig. 2). Indeed, TNFα and other inflammatory cytokines have been shown to induce autophagy through nuclear factor κB (NFκB) activation through an inhibitor of κB kinase α (IKKα)-dependent mechanism. This is particularly interesting because high circulating levels of IL-6 in humans with cancer correlate with a more advanced stage, poor prognosis and cachexia.^45,46^

## Combined inhibition of autophagy and ERK/MAPK signalling

The concept of combining inhibition of autophagy and inhibition of the KRAS-oncogene-driven signalling pathway emerged from the results of many studies that have shown a link between increased ERK/MAPK signalling and autophagy.^47^ Mechanistic studies suggest that ERK/MAPK signalling in fact negatively regulates autophagy but might also create a state of dependence on autophagy due to an altered metabolism.^23,24^ In this scenario, inhibition of ERK/MAPK signalling further increased autophagy but also increased sensitivity to autophagy inhibition. ERK/MAPK signalling can modulate autophagy/lysosomal function at several levels: inhibition of ERK/MAPK signalling directly activates 5′ AMP-activated protein kinase (AMPK), a master regulator of cellular energy homoeostasis, which can promote autophagy, and also increases the nuclear retention of TFEB, which co-ordinates the transcription of autophagy/lysosome genes (as mentioned previously).^22^ This is important for PDAC, in which ERK/MAPK activation is a near constant, in most cases due to activating mutations of KRAS.

### Inhibiting ERK/MAPK signalling creates autophagy dependence

Three groups concomitantly reported the efficacy of dual ERK/MAPK pathway and autophagy inhibition in KRAS-driven tumours.^23,24,48^ Two of these studies, which specifically addressed the efficacy of this combination in PDAC, showed that treatment with MAPK pathway inhibitors (inhibitors of either ERK/MAPK or MEK) synergised with autophagy inhibition. The study by Der and colleagues showed that the induction of autophagy upon inhibition of ERK/MAPK signalling is mediated by, and rescues cells from, the decrease in glucose metabolism secondary to ERK/MAPK inhibition.^23^ Furthermore, ERK/MAPK pathway inhibition (either by knockdown of KRAS or by pharmacological inhibition of ERK/MAPK) led to changes in mitochondrial function and decreased mitochondrial biogenesis. These changes contribute to create a state of autophagy dependence, which can be therapeutically targeted. This concept was further validated in several pancreatic cancer cell lines using different methods to inhibit ERK/MAPK signalling (shRNA-mediated knockdown of KRAS and pharmacological inhibition of ERK/MAPK and MEK) as well as different strategies to inhibit autophagy (ATG5 and ATG7 knockdown and chloroquine), as well as in patient-derived xenografts.^23^ The second group used various pharmacological inhibitors to show that blocking different nodes of the RAS–RAF–MEK/ERK signalling pathway increased spontaneously elevated autophagy in PDAC cell lines and that, again, targeting autophagy with chloroquine and hydroxychloroquine was synergistic with ERK/MAPK inhibition. The authors confirmed this synergy in vivo using the MEK inhibitor trametinib together with the inducible dominant-negative allele of ATG4B in xenografted cell lines.^24^ Finally, the synergistic effect of trametinib and chloroquine was further confirmed in patient-derived xenograft PDAC models as well as in NRAS-mutated melanoma and BRAF-mutated colorectal cancer models.^24^ One patient with advanced, chemotherapy-resistant PDAC treated with trametinib combined with hydroxychloroquine showed a partial response.^24^ Based on these promising results, the authors initiated a clinical trial investigating the combination of hydroxychloroquine and trametinib in patients with advanced PDAC pretreated with at least one line of chemotherapy (NCT03825289).

In the third study, investigators showed that co-targeting RAF (at least BRAF and CRAF) in combination with ATG7 had the highest toxicity ratio in KRAS-mutant versus KRAS WT cells (in both colorectal and PDAC cell lines).^48^ In addition, their experiments using pharmacological RAF inhibitors and siRNA-mediated knockdown of ATG7 showed increased tumour cell apoptosis compared with the use of RAF inhibitors alone.^48^ Taken together, these studies, as well as others conducted in other models,^49^ suggest that co-targeting the ERK/MAPK pathway and autophagy is synergistic in KRAS-mutant cancers, and suggest a potential new therapeutic approach for several difficult-to-treat cancers.

## Autophagy and metabolism

Autophagy and cell metabolism are two tightly connected cell processes that are difficult to study separately. One of the key aspects of tumour metabolism is the preferred use of aerobic glycolysis to produce lactate, rather than the oxidation of glycolytic pyruvate that occurs in differentiated cells. From the energy production aspect, the metabolism of glucose to lactate generates only two ATPs per molecule of glucose, whereas oxidative phosphorylation generates up to 36 ATPs upon complete oxidation of one glucose molecule. However, although aerobic glycolysis might not seem very efficient, the preference for this route of glucose metabolism can be explained in part by the use of glucose and lactate as building blocks for cell components (nucleic acid, proteins and membrane lipids) rather than just as an energy source. With this in mind, it is easy to see why autophagy, which uses damaged or unnecessary cell components and organelles to generate new building blocks, also plays an important role in cancer and how it is connected with tumour metabolism.^50^ As an example, AMPK and mTOR are major autophagy regulators that serve as energy and nutrient sensors, respectively.

### PDAC is associated with increased metabolism and autophagy

Pancreatic tumours, at least at their primary site (as opposed to metastases), are hypoxic and nutrient-poor, which might explain the high level of basal autophagy in these tumours. Furthermore, the activation of oncogenes, which often leads to increased proliferation, leads to an increase in tumour metabolism. This might be one of the reasons that autophagy is important for sustaining the survival and proliferation of KRAS-mutant tumours. This is exemplified in studies conducted in genetically engineered mouse models of KRAS-mutant lung tumours, in which genetic ablation of autophagy leads to the increased production of reactive oxygen species (ROS), lower energy levels, and a fall in the levels of nucleotide pools during starvation. In this model, therefore, autophagy sustains tumour metabolism by preventing fatal nucleotide depletion and energy crisis during starvation.^51^ In PDAC cell lines, inhibition of autophagy leads to a decreased oxygen consumption rate, potentially indicating decreased mitochondrial oxidative phosphorylation.^52^ Viale et al.^53^ have shown in an inducible model of KRAS signalling that resistance to KRAS-targeting therapy might be driven by a subpopulation of tumour cells that rely on oxidative phosphorylation for survival instead of the classic Warburg effect. Oxidative phosphorylation is highly dependent on mitochondrial respiration, and genes implicated in this phenomenon, as well as autophagy- and lysosome-related genes, were upregulated in surviving cells. As previously discussed, Perera et al. uncovered that, in these cells, the induction of autophagy is only one part of a broader transcriptional programme destined to supply tumour cells with adequate quantities of nutrients.^21^

Altogether, these data show that autophagy has a role in maintaining sufficient supplies of energy and nutrient to tumours via tumour-cell-autonomous, stromal and systemic autophagy. Combining inhibition of tumour autophagy and systemic caloric restriction to starve cancer cells has proved efficient in nude mice with kidney tumours but seems quite intriguing from a conceptual stand point because caloric restriction is known to lead to activation of systemic autophagy.^54^ In this sense, these results suggest that activation of systemic autophagy combined with inhibition of autophagy in tumours is more efficient than each of them separately. A possible explanation could be that caloric restriction causes a dependency on autophagy (which is higher in cancer cells given their high metabolic rate). Accordingly, this dependency would render cancer cells sensitive to autophagy inhibitors, explaining the observed effect.

## Autophagy and the anti-tumour immune response

As previously mentioned, the activity of programmed cell death 1 receptor (PD-1)/programmed cell death ligand 1 (PD-L1)- and cytotoxic T-lymphocyte-associated protein 4 (CTLA4)-blocking agents against PDAC is limited and the activity of novel combinations of immunotherapeutic agents reported so far has been at best disappointingly low in patients with microsatellite stable PDAC.^55,56^ The outcome of autophagy modulation to treat cancer is the result of a complex combination of the effect of autophagy on tumour cells and cells of the microenvironment, which includes immune cells. In addition, the interaction of various cell populations such as macrophages and fibroblasts, which both play a critical role in the immune context of PDAC, and T cells, renders deciphering the exact role of autophagy even more challenging. Data regarding the contribution of autophagy in immune cells in PDAC models are still limited, so we will also discuss some interesting observations made in other tumour models.

### Autophagy and immune cell function

Autophagy plays a major role in the differentiation and homoeostasis of immune cells (reviewed extensively elsewhere^57,58^), being required for the development and maturation of most immune cell types of both myeloid and lymphoid lineage. As with other studies on autophagy there is considerable heterogeneity with respect to the cellular phenotypes reported following the knockout of different autophagy genes. In myeloid cells, autophagy has been shown to play a key role in the survival of haematopoietic stem cells in the hypoxic bone marrow niche, but also seems to be critical for the maturation of monocytes to macrophages and the maintenance of macrophage differentiation.^59,60^ Autophagy plays an important role in LC3-mediated phagocytosis,^61^ and restrains dendritic-cell-activation, and thus their role in T-cell activation and maturation.^62^ The process of autophagy also contributes to antigen presentation by both MHC class I and class II molecules, by stabilising pathogen-containing phagosomes for prolonged MHC II antigen processing, which is critical for adequate CD4 T-cell stimulation.^58,63^

Autophagy also plays an important role in cells of the lymphoid lineage—for example, in the maintenance and functions of T cells, such as T-cell receptor (TCR) function, the modulation of regulatory T-cell functions, maintenance of long-lived cell populations such as memory T cells, and in the thymic maturation and selection process.^57^ Interestingly, at the whole animal level, one of the effects of inhibiting autophagy is lymphopenia. Being highly dependent on autophagy, regulatory T cells are one of the populations that are most vulnerable to autophagy inhibition/depletion. The effects of autophagy on mature immune cells, however, are much more subtle and variable across models and studies. Mitochondria-specific autophagy, called mitophagy, is required for the long-term survival of memory T cells.^57^ Thus, again, the role of autophagy cannot be categorised as either immunosuppressive or immune-stimulatory, but rather as a process required for homoeostasis and normal function. As such, a precise understanding of the molecular mechanisms involved will be necessary to leverage autophagy as a therapeutic target.

### Autophagy and the immune response in tumours

Yang et al. used a genetically engineered mouse model of pancreatic cancer expressing the inducible dominant-negative form of ATG4B (ATG4B^C74A^) to show that the tumour regression induced by inhibition of autophagy was at least in part immune mediated.^40^ The authors observed an increase in the infiltration of macrophages (but not T cells) in the tumours upon inhibition of autophagy, and this was associated with increased anti-tumour activity. On the contrary, however, several groups have reported, using various models and experimental settings, that activation of autophagy reduced the tumour-promoting capacity of tumour-associated macrophages (TAMs). In their study, Starobinets et al.^64^ showed no significant effect of inhibiting autophagy in tumour cells on the adaptive immune response in various cancer models. Furthermore, they demonstrated that systemic inhibition of autophagy using chloroquine had no effect on tumour growth and T-cell response in B16 (melanoma) or 4T1 (breast cancer) models in immunocompetent hosts. These findings contradict those of Michaud et al.,^65^ who previously showed that an autophagy-dependent immune response increased the activity of some cytotoxic agents that were able to induce immunogenic cell death, including some currently used in the treatment of PDAC (i.e. oxaliplatin). Although the precise mechanism of the observation by Michaud et al.^65^ remains unclear, the same team later reported this effect to be due to the production of type I interferon by tumour cells, which induced a vaccine-like immune reaction.^66^ This team also examined the effect of caloric restriction mimetics, most notably hydroxycitrate, which mimic nutrient deprivation, on autophagy and on the efficacy of immunogenic-cell-death-inducing chemotherapy. In their model, hydroxycitrate induced autophagy, which led to improved efficacy of the topoisomerase inhibitor mitoxantrone. Interestingly, this effect was not only dependent on autophagy, but also required T-cell activity, suggesting a link between autophagy and the immune response. In a model of Kras-induced lung cancer, these authors also observed a reduction in the number of regulatory T cells, which paralleled the tumour-preventive activity of hydroxycitrate.^67^

### Autophagy and immunotherapy

Autophagy has already been shown to be required for the tumour response to various anti-cancer therapies,^68,69^ including chemotherapy and radiotherapy, which can induce autophagy on their own and can potentiate the effect of immunotherapy.^70^ As autophagy plays an important role in antigen processing and presentation by MHC class II molecules, it might enhance the efficacy of currently used checkpoint inhibitors. Several reports, although none specifically in PDAC, have shed light on the role of autophagy in anti-tumour immunity. Pietrocola et al.^67^ convincingly showed, in a fibrosarcoma model, that the synergistic effect of caloric restriction mimetics and immunogenic-cell-death-inducing cytotoxic agents is dependent on CD8 T cells. This same team further showed dramatic synergy between immunogenic-cell-death-inducing cytotoxic agents, caloric restriction mimetics and immune checkpoint inhibitors,^71^ thereby suggesting that activating, or further enhancing, autophagy might help anti-cancer therapy (i.e. chemotherapy and immune checkpoint) to eradicate established tumours. Although the authors clearly demonstrated the autophagy- and immune-cell-dependence of their observations, the molecular mechanisms remained unclear. Two studies, published in 2018 and 2019, investigated the molecular connection between autophagy and T-cell function. Vodnala et al.^72^ showed that increased extracellular potassium levels (which can arise in tumours due to spontaneous necrosis) maintain CD8 T-cell stemness by limiting nutrient uptake, thereby inducing autophagy and reducing histone acetylation, which generates CD8+ T cells with enhanced persistence. Interestingly, these effects of increased extracellular potassium concentration can be induced by the addition of hydroxycitrate (used by Mariño et al.).^73^ In addition, Shulka et al. identified an association between the expression of the melanoma antigen gene (MAGE) A family of classic cancer-testis antigens and resistance to anti-CTLA4 therapy in patients with melanoma: MAGE A proteins contribute to the formation of a ubiquitin ligase complex, which then mediates the ubiquitination and proteasomal degradation of key autophagy proteins. This suggests that suppression of autophagy is a specific resistance mechanism to CTLA4 in humans^74^ (as this correlation was not observed in anti-PD1-treated patients) and that restoring autophagy might confer a potential synergistic effect in conjunction with anti-CTLA4 therapy. DeVorkin et al. reported increased anti-tumour efficacy, despite decreased overall T-cell infiltration, with autophagy-deficient T cells generated using genetic ablation of key autophagy genes (ATG5, ATG16L and ATG14) and bone marrow transplant (to specifically study the effect in leucocytes/bone-marrow-derived cells).^75^ Interestingly, the observations of the epigenetic landscape of T cells in this study, in terms of autophagy induction and inhibition of T-cell maturation, mirror those made by Vodnala et al., which is reassuring. However, uncertainty remains regarding the optimal stage of T-cell maturation (stem-like in Vodnala et al. or effector-memory in DeVorkin et al.) required for anti-tumour immunity, although this might vary from one model to another.

Other studies have shown a potential synergy of immunotherapy with inhibition of autophagy. For example, Liang et al.^76^ showed synergy between high-dose IL-2 and chloroquine in a model of liver metastases, an approach that enabled the toxicity of IL-2 to be limited. The mechanistic basis for the differential effect of autophagy on tumour tissue compared with normal tissue was not reported in this study.

Recently, MHC-I molecules were shown to be sequestered in autophagosomes in pancreatic cancer cells, which prevents recognition by T-cells and contributes to immune escape of pancreatic cancer cells.^77^ In this study, the authors, who use a dox-inducible ATG4B^DN^ model, show that inhibition of autophagy increases MHC-I expression at the cell surface and antigen-specific T-cell activation, which translate in vivo into increased T-cell infiltration. Furthermore, inhibition of autophagy (ATG4B^DN^) enhanced sensitivity to dual immune check point inhibition (anti-CTL4 + anti-PD-1). It should be noted however that (i) the same team using the same model was unable to show increased T-cell infiltration upon autophagy inhibition in a previous study^40^ and (ii) the extent of sensitisation to immune check point, although sufficient to slow down tumour growth, is not sufficient to elicit tumour eradication.^77^

## Autophagy in PDAC: translational findings

### Autophagy as a prognostic biomarker in PDAC

In tumours, the status of several autophagy proteins, such as LC3b, ATG5, Beclin1 and its interacting proteins high mobility group Box 1 (HMGB1) and the Bcl-2-family, mostly assessed using immunohistochemistry, has been evaluated as a marker in several tumour types. Indeed, some studies have shown that expression or overexpression of these autophagy proteins is linked with a better prognosis in lung cancer,^78^ colorectal cancer^79^ and breast cancer.^80,81^ The data are, however, scarcer for PDAC. One study showed that Beclin1 overexpression correlated with a more advanced stage but was not significantly associated with overall survival in multivariate analysis.^82^ In another study investigating the prognostic value of autophagy markers, the overexpression of Beclin1 and LC3-II was associated with decreased survival, but there was no multivariate analysis included in this study, which limits its interpretability.^83^ Partial loss of ATG5 was reported to be associated with worse prognosis in patients with PDAC; however, the prognostic effect in this study is marginal and again was not assessed in multivariate analysis.^39^ Thus, the value of autophagy proteins as biomarkers in PDAC currently remains largely unexplored. Furthermore, difficulties in adequately assessing autophagy activation or autophagic flux in clinical samples (in paraffin embedded samples, for example) is an obstacle to adequately assessing the value of autophagy as a prognostic or predictive biomarker.

### Pharmacological modulation of autophagy

Autophagy-deficient (Atg5^−/−^) mice do not survive the neonatal starvation period—the period between birth, when the nutrient supply from the placenta is interrupted, and the restoration of supply through milk nutrients—and although this has been attributed to a deficit in amino acid supply, it has been shown that these mice can be salvaged by Atg5 re-expression in the brain.^84,85^ To study the effects of autophagy ablation in adult mice has thus required the development of specific models. Toxicity (most importantly, neurodegeneration) observed in these models of genetic ablation of key autophagy genes raises concerns regarding the safety of the approach: with higher susceptibility to infection, glucose homoeostasis imbalance, neurodegeneration, tissue damage (muscle, liver and pancreas) and heart failure being among the recorded outcomes.^37,86^ These observations are not surprising given the important role of autophagy in cellular homoeostasis and stress response, and together suggest that complete ablation of autophagy is unlikely to be sustainable in humans, either. Conversely, moderate activation of autophagy has been shown to have a positive impact on lifespan in some models,^87^ but was associated with increased toxicity, especially cardiac toxicity, in others.^88^ In general, non-pharmacological autophagy-inducing behaviours, such as exercise^89^ and caloric restriction through fasting/dieting,^11^ have been shown to have a favourable effect on general health.

Unlike genetic manipulations, pharmacological inhibition is rarely perfectly specific, complete and definitive. Thus, the effects generated by genetic ablation of major autophagy genes are unlikely to be exactly replicated with pharmacological inhibition. The specificity of autophagy inhibition using pharmacological tools remains an issue that is rendered even more complex by the multiple autophagy-independent roles of many autophagy-related gene products, although some of these autophagy-independent roles are dependent on scaffolding properties rather than catalytic activities,^90^ which might thus be amenable to specific pharmacological modulation^91^ The following information aims to provide a brief overview of this field, although there are many more comprehensive reviews on the pharmacological modulation of autophagy.^91,92^

#### Inhibiting autophagy

Chloroquine and hydroxychloroquine have long been used to inhibit autophagy in both preclinical and clinical studies, but because both of these compounds also target lysosomal functions, they not only inhibit the disposal of autophagosomes, but also the degradation of endosomes, as well as impairing vesicular trafficking. Furthermore, several studies have shown that the anti-tumour activity of chloroquine and hydroxychloroquine can be uncoupled from their autophagy-inhibitory properties.^35,93^ A number of other autophagy inhibitors, such as Lys05 family,^94,95^ ROC-305^96^ and GNS561,^97^ which are at different stages of clinical development, also target lysosomes. In fact, hydroxychloroquine and other lysosomotropic drugs probably also inhibit other cell mechanisms that converge on the lysosome, such as micropinocytosis, which could lead to the inhibition of tumour growth,^98^ or might induce lysosomal permeation, which could have a potent anti-tumour effect independent of the effect on autophagy.^99^ Because chloroquine and hydroxychloroquine have been approved for a long time and are readily available, several studies have investigated these compounds in patients with pancreatic cancer (see below).

Most of the proposed autophagy targets for pharmacological intervention are kinases which, as a class, have been shown to be readily targetable with small molecules, with ever-increasing specificity and potency, since the 1990s.^100^ Few of these compounds have progressed to clinical use, however, mainly because of the uncertainties and controversies regarding the effect of targeting autophagy in cancer. The ULK1 kinase complex, which comprises the serine/threonine kinase ULK1 (ATG1), ATG7 and FIP200 (Box 1), is activated in response to nutrient deprivation and serves as a critical initiator of starvation-induced autophagy. Several specific inhibitors of ULK1 have been identified (SBI-0206965),^101,102^ but none has yet progressed to clinical trials. Similarly, inhibitors of ATG7 have been described but none has yet progressed to clinical use.^103^ The lipid kinase VPS34, also required for the initiation of autophagy, is a member of the phosphatidylinositol 3-kinase (PI3K) family, which has been the focus of intense research efforts resulting in the development over the past 20 years of several general (3-methyladenine, wortmannin, LY294002) and isoform-specific inhibitors (e.g. alpelisib, idelalisib). New specific VPS34 inhibitors (e.g. SAR-405) have been described in the past decade, but the development of some of them has been stopped after controversies around the effect of autophagy in cancer.^104–106^ However several companies are still developing VSP34 inhibitors for potential use in combination with targeted and immune therapies.^107^

ATG4 is not a kinase but a cysteine protease; it contributes to autophagosome formation by processing LC3/ATG8 paralogues from their precursor form to their active form (LC3-I) by revealing the phosphatidylethanolamine conjugation site, and is also responsible for the deconjugation (delipidation) process that allows the recycling of LC3. Although the dominant-negative form (ATG4B^C74A^) sequesters LC3, ATG4B knockout only leads to partial inhibition of autophagy owing to rescue by other ATG4 isoforms.^108^ Thus, the value of isoform-specific versus pan-ATG4 inhibition to target autophagy is still debated and will require additional research.^109^ Similar to other autophagy targets, several ATG4B inhibitors have been reported over the past decade, but none is yet in clinical development.^110,111^

#### Activating/enhancing autophagy

Although most of the current drug development efforts are aimed at inhibiting autophagy, activating autophagy could also have an anti-tumour effect on the whole organism by modulating the immune response. Extensive clinical experience is available from the mTOR inhibitor rapamycin and its derivatives (rapalogues), which have proven safe and active for the treatment of some diseases, although their precise mechanism of action remains uncertain.^112^ Interestingly, the most robust predictor of the clinical activity of rapalogues identified so far has been the loss of the mTORC1 regulators TSC1 and TSC2,^113–115^ but no direct correlation with the status of autophagy has yet been made. Furthermore, some preclinical studies show a synergistic cytotoxic effect of mTOR inhibitors and autophagy inhibitors, as opposed to the cytostatic effect of single-agent mTOR inhibitors.^105^ The explanation for this counterintuitive observation probably lies in the fact that the induction of autophagy might in fact be a survival response mechanism triggered by mTORC1 inhibition that limits the single-agent activity of these compounds,^105^ rather than a direct effect of mTORC1 on autophagy activation. Furthermore, rapalogues have been shown to be less efficient than nutrient deprivation at inducing autophagy.^26^ The clinical relevance of these findings has, however, yet to be demonstrated. Single-agent studies of mTOR inhibition (mostly with rapalogues) have failed to show any significant activity in humans with PDAC.^116^

Other autophagy activators acting via acetyl-CoA depletion, acetyl transferase inhibition or deacetylase inhibition, all included under the caloric restriction mimetic umbrella, have not demonstrated activity in clinical trials in PDAC so far.^91,117^ Many companies are exploring the field of autophagy modulation,^117^ and a more detailed list of potential autophagy modulators can be found in other reviews.^91^

## Clinical trials involving autophagy modulation in patients with PDAC

Only a handful of autophagy-modulating compounds are available for clinical use: chloroquine and hydroxychloroquine as inhibitors of autophagy, and the mTOR inhibitors everolimus and temsirolimus as activators of autophagy, have been tested in clinical studies in pancreatic cancer patients.

### Inhibiting autophagy using chloroquine and hydroxychloroquine

Only one Phase 2 study has investigated the activity of single-agent hydroxychloroquine in patients with previously treated metastatic PDAC. In this study, 20 patients received hydroxychloroquine at 400 mg (*n* = 10) or 600 mg (*n* = 10) twice daily. LC3-II levels in peripheral lymphocytes were assessed as pharmacodynamic markers of autophagy inhibition. No significant safety findings were observed, but no responses were observed either, and only two patients were progression-free at 2 months.^118^ Autophagy inhibition, as demonstrated by an increase in LC3-II levels in peripheral lymphocytes, was inconsistent.

As autophagy is believed to be a mechanism of resistance to cytotoxic chemotherapy, combining chloroquine or hydroxychloroquine with chemotherapy seems a logical step. In a Phase 1 study of gemcitabine combined with chloroquine, nine patients received weekly gemcitabine at standard doses (1000 mg/m²) together with weekly chloroquine at ascending doses.^119^ No dose-limiting toxicity was seen and three patients had a partial response (33%), but median progression-free survival and overall survival (4 and 7.6 months, respectively) were within expected values for treatment with gemcitabine alone. In a Phase 1b/2 study reported by Boone et al., patients with ‘borderline resectable’ PDAC were treated with two doses of gemcitabine (1500 mg/m^2^) at 2-week intervals together with once daily hydroxychloroquine given in most patients at 1200 mg for 31 days, prior to surgery.^120^ No radiographic assessment of response was carried out in this study, but no pathological complete response was seen on surgically resected samples. The majority of patients showed a decline in the levels of the marker CA19.9 following preoperative treatment, and 65% of patients had a more than 50% increase in LC3-II staining on peripheral blood mononuclear cells, which was used as a surrogate marker for autophagy inhibition. These patients showed improved survival and progression-free survival (PFS) compared with patients who showed a less than 50% increase in LC3-II staining. No unexpected safety signal was reported. Although no definitive conclusion regarding the benefit of adding chloroquine or hydroxychloroquine can be drawn from these two studies because of the limited number of patients and lack of randomisation, the correlation of increased LC3-II staining on peripheral blood mononuclear cells with PFS is intriguing because it suggests that the lower efficacy seen in other patients might be due to a lack of pharmacodynamics activity of hydroxychloroquine in these patients (possibly due to lower exposure).

In a 2019 randomised Phase 2 trial, Karasic et al.^121^ compared gemcitabine and nab-paclitaxel (one of the current standard chemotherapy options for first-line advanced pancreatic cancer^122^) with or without hydroxychloroquine in 112 patients. Although this study failed to reach its primary endpoint (improved overall survival rate at 12 months), the authors reported a significant increase in the response rate (from 21% to 38%, *P* = 0.047), but this did not translate into increased PFS. There was a manageable increase in chemotherapy-related adverse events in the hydroxychloroquine-treated group (mostly myelosuppression and gastrointestinal symptoms), together with some hydroxychloroquine-specific effects such as visual changes and neuropsychiatric symptoms. The genomic analyses (conducted in only 40% of patients with sufficient archived material) showed no significant correlation between tumour genomic alterations (including *KRAS* mutations and *TP53* alterations) and the response to hydroxychloroquine. Based on these results, the authors proposed to use hydroxychloroquine combined with preoperative chemotherapy, a setting in which the increase in response rate (which remains to be confirmed in additional studies) might increase curative resection rates. Interestingly, an increase in pathological response rate was reported in an interim analysis of a study investigating the addition of hydroxychloroquine to preoperative gemcitabine and nab-paclitaxel (although here, again, no pathological complete response was seen;^123^ NCT01978184).

### Activating autophagy

Although most of the ongoing efforts are aimed at inhibiting autophagy in pancreatic cancer, the question remains, as discussed previously, as to which strategy is best: to inhibit or to activate autophagy. The activation of autophagy via rapalogue-mediated mTOR inhibition has shown interesting results in preclinical studies in PDAC models through inhibition of the PI3K–AKT signalling pathway, but these results have not translated into significant clinical activity as single agents in humans.^116,124^ One explanation could be that mTORC1 inhibition induces a feedback loop leading to the phosphorylation of AKT.^125^ New agents or combinations that avoid this feedback loop might be of interest in PDAC,^126^ but the fact that mTORC1 has numerous phosphorylation targets other than the ULK1 complex needs to be emphasised, which limits the conclusions that can be drawn regarding autophagy activation by rapalogues and other mTOR inhibitors.

## Discussion and future directions

Autophagy has been a longstanding putative target for the treatment of PDAC. Our understanding of autophagy has increased drastically in the past decade thanks to new genetically engineered mouse models of cancer and the advent of high-throughput tools. The emerging picture is clearly more complex than initially anticipated with not only an increasing appreciation of the role and mechanisms of selective autophagy, but also the identification of autophagy-independent functions of major components of the autophagy machinery. A set of reliable and widely accessible compounds targeting some of the key autophagy components is, however, still needed to deepen our understanding of the consequences of pharmacological modulation of autophagy and help its translation to human use. On the clinical side, trials investigating the biological effects of currently available autophagy-modulating compounds will improve our understanding of these effects in humans and will enable the establishment of a correlation with preclinical models, thus improving their predictive value.

Autophagy is considered to be a mechanism by which tumour cells maintain their high metabolic levels in a nutrient-poor environment. However, it also appears that tumour cells can use autophagy from surrounding cells to maintain their supply of essential nutrients. Data also suggest that, even in tumour cells, autophagy levels are modulated by the nutrient and energy balance. In that sense, time-dependent modulation of autophagy might be an interesting option to upset tumour homoeostasis. Indeed, autophagy helps tumour cells cope with multiple stresses, including maintaining energy homoeostasis and nutrient pools created by unregulated proliferation and adverse microenvironmental conditions such as hypoxia, low pH and decreased nutrient supply. This may create a state of dependence of tumour cells towards continuous activation of autophagy. Although the general hypothesis was that inhibition of autophagy was the way forward based on the fact that the autophagic flux is described as being constitutively high in PDAC, the effects of autophagy modulation on the immune system and the identification of feedback loops question this theory and might pave the way for new therapeutic concepts. Overall, the fact that autophagy has been described to be both tumour-suppressive and tumour-promoting in PDAC does not mean that it cannot be therapeutically modulated. The vast majority of evidence points toward inhibiting autophagy in PDAC, and results from the combined inhibition of the ERK/MAPK pathway and autophagy have led to the initiation of several clinical trials investigating various ERK/MAPK pathway inhibitors in combination with hydroxychloroquine (NCT04145297; NCT03825289; NCT04132505). The effect of modulating autophagy on the anti-cancer immune response will require evaluation in humans, as mouse models have so far shown limited predictive value due to significant differences between murine and human immune system functions and the inherent limitations of carcinogen-induced and genetically engineered models.

Research to better understand autophagy in humans is still hampered by limitations in our current tools to assess this process. Thus, the development of simple, robust and reliable methods to assess autophagy in human samples (blood and tumour) will be a major step towards designing clinical trials that assess autophagy modulation and for biomarker-based patient selection.

## Data Availability

No new data was generated.
